# Phenolic Compounds Content and Genetic Diversity at Population Level across the Natural Distribution Range of Bearberry (*Arctostaphylos uva-ursi*, Ericaceae) in the Iberian Peninsula

**DOI:** 10.3390/plants9091250

**Published:** 2020-09-22

**Authors:** Esther Asensio, Daniel Vitales, Iván Pérez, Laia Peralba, Juan Viruel, Celia Montaner, Joan Vallès, Teresa Garnatje, Ester Sales

**Affiliations:** 1 Departamento of Química Analítica, Instituto de Investigación en Ingeniería, Universidad de Zaragoza, Centro Politécnico Superior, Edificio Torres Quevedo, María de Luna 3, 50018 Zaragoza, Spain; estherac@unizar.es; 2 Institut Botànic de Barcelona (IBB, CSIC-Ajuntament de Barcelona), Passeig del Migdia s.n., 08038 Barcelona, Spain; daniel.vitales@ibb.csic.es (D.V.); ivanpe04@ucm.es (I.P.); lperalpa7@alumnes.ub.edu (L.P.); tgarnatje@ibb.csic.es (T.G.); 3 Laboratori de Botànica (UB)—Unitat Associada al CSIC, Facultat de Farmàcia i Ciències de l’Alimentació—Institut de Recerca de la Biodiversitat (IRBio), Universitat de Barcelona, Avda. Joan XXIII 27-31, 08028 Barcelona, Spain; joanvalles@ub.edu; 4 Royal Botanic Gardens, Kew, Richmond TW9 3DS, UK; j.viruel@kew.org; 5 Departamento of Ciencias Agrarias y del Medio Natural, Instituto Universitario de Ciencias Ambientales, Universidad de Zaragoza, Escuela Politécnica Superior, Ctra. Cuarte s.n., 22071 Huesca, Spain; cmonoti@unizar.es

**Keywords:** arbutin, genetic and phytochemical variability, genome size, haplotypes, natural antioxidants

## Abstract

Bearberry (*Arctostaphylos uva-ursi*) is a medicinal plant traditionally employed for the treatment of urinary tract infections due to high contents of arbutin (hydroquinone β-D-glucoside), which is now mainly used as a natural skin-whitening agent in cosmetics. Bearberry has also been proposed as a natural antioxidant additive due to the high contents of phenolic compounds in leaves. We studied the variation on phenolic compounds in 42 wild populations of bearberry, aiming to elucidate if intrinsic biological, climatic, and/or geographic factors affect phenolic contents across its natural distribution in the Iberian Peninsula. Bearberry leaves were collected during autumn over a three-year period (2014–2016) in populations across a latitude and altitude gradient. Methanolic extracts showed a wide range of variation in total phenols content, and different phenolic profiles regarding arbutin (levels of this major constituent varied from 87 to 232 mg/g dr wt), but also catechin and myricetin contents, which were affected by geographic and climatic factors. Moderate levels of variation on genome size—assessed by flow cytometry—and on two plastid DNA regions were also detected among populations. Genetic and cytogenetic differentiation of populations was weakly but significantly associated to phytochemical diversity. Elite bearberry genotypes with higher antioxidant capacity were subsequently identified.

## 1. Introduction

The synthesis of plant specialized metabolites varies in time (i.e., ontogeny, phenology, and induced defenses), and space, as it plays a crucial role in plant adaptation to environmental conditions, while also genetic variation accounts for chemodiversity [1]. Among these compounds, phenols have a diverse array of mono- and polymeric structures that fulfil a broad range of physiological roles [2]. The biosynthesis of specialized metabolites is greatly affected by environmental factors such as temperature, precipitation, or solar radiation, which are in turn often subjected to latitudinal, longitudinal, or altitudinal gradients. Particularly, phenolic compounds accumulation is a general response to enhanced levels of UV-B (280–315 nm) radiation. Specific compound variation has been reported in plants growing in the Mediterranean region during the summer and at high altitudes, where a higher incidence of UV-B occurs and cinnamic acids and flavonoids showed the highest UV absorption rates [3]. 

Arbutin (hydroquinone β-D-glucoside) is a simple phenol compound with a restricted occurrence in the leaves of some species belonging to genera such as *Arbutus*, *Arctostaphylos*, *Pyrus*, or *Vaccinium*. The main natural source of arbutin, the bearberry (*Arctostaphylos uva-ursi* (L.) Spreng.) has been used for centuries to treat urinary tract infections and other renal diseases [4], and herbal formulations are still prepared nowadays [5]. Over the last years, the spectrum of applications of arbutin has broadened, mostly as a natural skin-whitening agent in the cosmetic industry [6] and in clinical therapies due to its antioxidant, antibiotic, anti-inflammatory, and antitumor properties [7]. Consequently, there is an increasing interest in finding additional natural sources of arbutin, as well as biotechnological processes that can replace chemical synthesis [8,9]. In this context, in vitro production of arbutin using *Datura inoxia* cell cultures has reached the pilot scale [10].

In addition to arbutin, other phenolic compounds contribute to *A. uva-ursi* active properties, such as flavonoids and tannins [11,12], from which catechin and corilagin are the most relevant in leaves, respectively [13]. Since in the food industry natural compounds as plant phenols are replacing synthetic antioxidant preservatives [14]. *A. uva-ursi* has been used as an additive, especially in meat products [15,16,17,18,19] but also in active packaging [20]. Finally, the potential of *A. uva-ursi* as a source of tanning agents for the leather industry has been recently proposed [21]. 

The “bearberry leaf” monograph of the European Medicines Agency [5] proposed a minimum of 7% of arbutin content in dried leaves as requirement for herbal preparations. Studies performed over the past decades described arbutin contents in bearberry leaves varying from 0% to 18%, explained by analytical procedures, natural variability, growth conditions and harvesting date [4]. Arbutin content is usually determined by high performance liquid chromatography [4,12,22,23,24], and previous studies detected higher arbutin contents in bearberry plants collected in fall than in those collected in spring [4]. Diverse bearberry chemotypes have been described, associated with the geographical distribution and to the intraspecific differentiation in subspecies of *A. uva-ursi*. The most striking phytochemical variation reported is the absence of arbutin in *A. uva-ursi* subsp. *stipitata* Packer and Denford, but also differences in methyl-arbutin, ellagic acid, or myricetin contents have been referred. Quercetin is also present in bearberry leaves, in which both aglycones, as well as their 3-O-glycosides are the more commonly found flavonoids [4]. However, most of these studies were performed several decades ago, and nowadays intraspecific variation in bearberry is considered to be continuous. The 14 subspecies described, including one from Spain (*A. uva-ursi* subsp. *crassifolius* (Braun-Blanq.) Rivas Mart. ex Torre, Alcaraz and M.B. Crespo), are currently non-accepted names and synonymous of *A. uva-ursi* [25]. New investigations are therefore needed to improve our knowledge on the compounds actually present in the raw material harvested from different locations for industrial purposes, since the chemical profile of *A. uva-ursi* leaves has been described to include a diverse array of phenols, tannins, and flavonoids in more recent studies performed to investigate differences at the interspecific level [23,26,27].

Genetic (including cytogenetic) diversity at population level promotes adaptation to different environmental conditions [28]. The effects of polyploidization on the production of specialized metabolites in medicinal and aromatic plants were recently reviewed [29]. In this context, genome size is a largely used parameter in plant variability assessments, since intraspecific variation is often detected ([30], and references therein), correlated with many other biological traits, and playing a relevant role in evolutionary processes [31,32,33,34,35]. However, studies addressing the association between nuclear DNA amount (i.e. genome size) and phytochemical diversity are very scarce [36,37]. The relationship between phytochemical and genetic diversity has been assessed using nuclear but especially plastid DNA markers [38], particularly in medicinal [39] and cultivated [40] plant species. 

Bearberry is widely distributed throughout the circumboreal area, but most of the collected wild populations in Europe are located in the Eastern countries, Austria, Switzerland, Italy, and Spain. *Arctostaphylos uva-ursi* grows in the eastern mid part of Spain, in mountains, in altitudes ranging from 550 to 2350 m above sea level (a. s. l.). It is more common in the North, since populations located at lower latitudes are less frequent and usually have fewer individuals. The existing studies regarding the arbutin content variability in Spain referred values ranging from 8% in north-eastern populations [41] and 19% in Spanish plant material evaluated in a study performed in Germany [42]. Our study aims to elucidate factors, including genome size and genetic diversity, explaining phytochemical variation across the natural distribution of bearberry in Spain. Our results could contribute to the selection of plant material for pharmaceutical, cosmetic, and food industries.

## 2. Results and Discussion

### 2.1. Chemical Diversity Analysis

**Figure 1:**
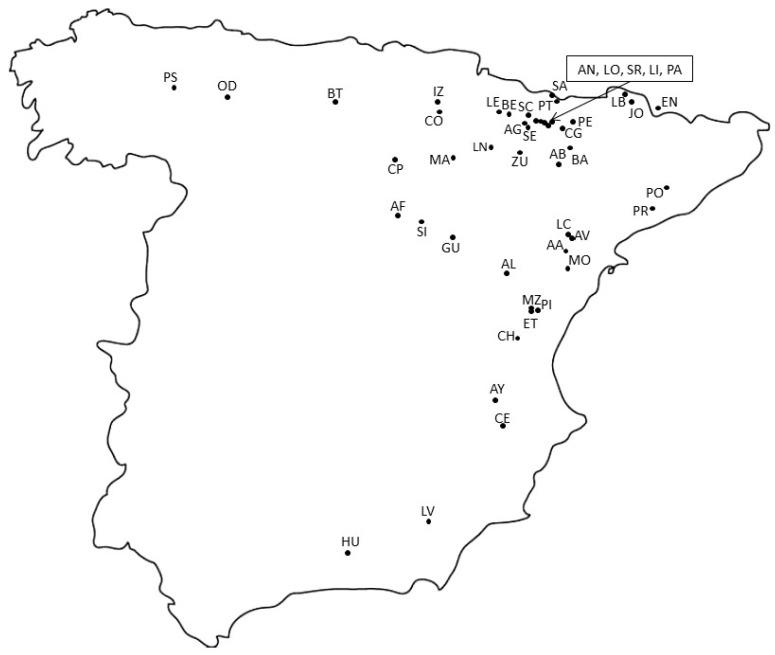
Location of the 46 natural populations of bearberry sampled in this study. Phytochemical analysis was performed using plant material from 42 locations, while genetic and cytogenetic studies were performed using plant material from 37 and 35 locations, respectively. Population codes are detailed in Table S1.

**Figure 2:**
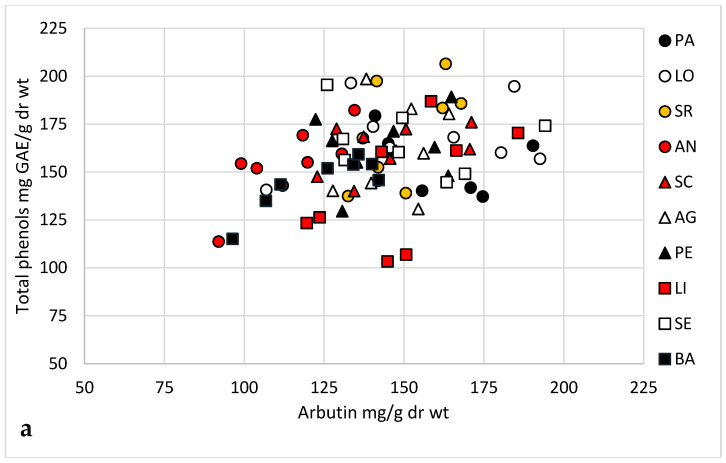

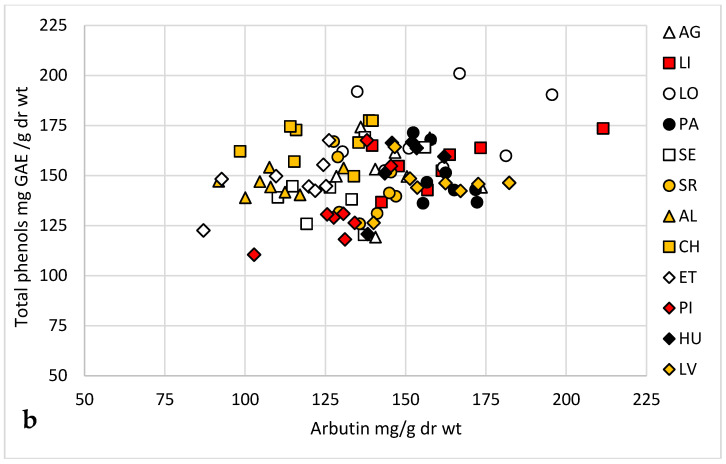
Total phenols (mg GAE/g dr wt) and arbutin (mg/g dr wt) content of leaves collected in 2014 from 80 bearberry plants growing in 10 natural populations of north Spain (**a**) and from 94 plants growing in 12 populations sampled in 2015 (**b**).

Bearberry leaves sampled from a total of 249 plants growing in 42 Spanish locations, and across a three year-period, 2014–2016 (Figure 1, Table S1), showed different dry weight (dr wt) percentages, ranging from 46.7 (LO) to 55.1% (CP), with an average of 50.1 ± 2.7% (data not shown). Methanolic extracts prepared from samples collected in 2014 and 2015 were employed for total phenols and arbutin content determinations. We observed a wide range of continuous variation for both parameters with significant differences (*p* < 0.001) among plants. Leaf extracts of 80 plants sampled in autumn 2014 (Figure 2a) showed total phenolic contents ranging from 103.3 ± 4.8 mg GAE/g dr wt (sample LI-4) to 206.4 ± 6.5 mg GAE/g dr wt (SR-2), while arbutin contents fluctuated from 92.0 ± 3.0 mg/g dr wt (AN-6) to 194.2 ± 5.6 mg/g dr wt (SE-8). Analysis of extracts prepared from leaves collected in 2015 from 94 plants (Figure 2b) also showed a wide phytochemical variation, from 110.5 ± 3.6 mg GAE/g dr wt (PI-4) to 200.9 ± 9.8 mg GAE/g dr wt (LO-4) in total phenolic contents, while arbutin contents ranged between 87.1 ± 0.4 mg/g dr wt (ET-2) and 211.5 ± 5.9 mg/g dr wt (LI-2). Furthermore, irrespective of the year of collection, significant differences in total phenols and arbutin contents were detected among bearberry plants growing in the same location for most of the populations (data not shown). Arbutin levels were significantly correlated with total phenols contents in bearberry leaf extracts, since we estimated significant Pearson coefficients of 0.332 (*p* = 0.003) for 2014, and 0.289 for 2015 data (*p* = 0.005). Arbutin contents of the 48 plants sampled in both years did not vary significantly (*p* = 0.380).

**Figure 3:**
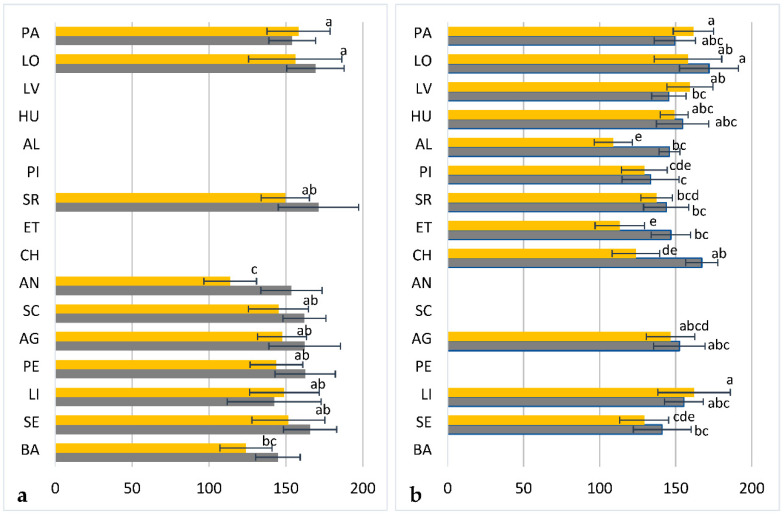
Mean total phenols (mg GAE/dr wt, grey bars) and arbutin (mg/g dr wt, yellow bars) contents of leaf extracts from bearberry plants collected in 2014 (**a**) and 2015 (**b**) in 16 natural populations of Spain located in a wide range of altitude, from 424 (BA) to 1410 m a. s. l. (PA). For each location, data are mean and standard deviation of eight plants analyzed in triplicate (except for HU, N = 6). Within each set of data, values followed by the same letter were not different according to Tukey test.

Despite the variability found within populations, and irrespective of the year of harvest, analysis of variance showed that arbutin contents of bearberry leaf extracts also depended (*p* < 0.001) on population location (Figure 3a,b), as occurred for total phenols content of bearberry leaves analyzed in 2015 (*p* < 0.001, Figure 3b). In contrast, significant differences in total phenols contents were not observed (*p* = 0.080) among the 10 populations sampled in 2014, which were located in a relatively small area, but distributed in a wide range of altitude, from 424 (BA population) to 1410 m a. s. l. (PA population). This variation in altitude was associated to differences in climatic conditions (Table S1), since locations at higher altitudes were characterized by lower mean temperatures (Pearson’s coefficient of correlation, r = −0.666, *p* < 0.001) and higher annual precipitations (r = 0.485, *p* < 0.001), which in turn resulted in lower global radiation levels (r = −0.390, *p* < 0.001). Furthermore, from this data set we estimated low but significant positive correlation between annual rainfall and total phenols contents of bearberry plants (Spearman’s coefficient rho = 0.256, *p* = 0.022), whereas from the 2015 data set, when we sampled populations in a larger area, we detected significant positive correlation between annual rainfall and arbutin content (rho = 0.246, *p* = 0.017). This significant correlation indicates that bearberry plants growing in northern locations and at relatively higher altitudes showed frequently higher arbutin contents (rho = 0.217, *p* = 0.035 and rho = 0.269, *p* = 0.009, respectively). As mentioned, these higher arbutin contents correlated significantly to higher total phenols contents of bearberry leaves, which were similar (on average 154.4 ± 19.6 mg GAE/g dr wt) to those found in aqueous extracts from other plant species employed as antioxidant additives in the food industry [43], such as rosemary (185.0 mg GAE/g dr wt), tea (149.3 mg GAE/g dr wt), or guava (154.4 mg GAE/g dr wt). These results are in agreement to previous references, and were corroborated in a study performed by Wrona et al. [20] using some of the Spanish bearberry leaf samples collected in 2015, for which higher antioxidant capacity was associated to higher arbutin contents. Elite bearberry genotypes could be also identified for this purpose, such as individuals 1, 4, 7, and 8 from the LO population, which accumulated on average 183.3 ± 16.4 mg GAE/g dr wt in the two years of study.

In order to further elucidate climatic and geographical factors affecting the patterns of phenolic variation, we performed a more exhaustive sample collection in autumn 2016: 140 bearberry plants growing in 29 locations, and we quantified five phenolic compounds (Figure S1a) that were identified by co-elution with standards in an acetonitrile/water gradient (Figure S1b). Analysis showed wide ranges of continuous variation for arbutin, caffeic acid, catechin, myricetin, and quercetin glucoside contents, with significant differences among plants that were also generally observed within populations (data not shown). For the major phenolic constituents, we found that arbutin contents ranged from 91.1 ± 5.0 (LB-2) to 232.4 ± 2.8 mg/g dr wt (PT-5), while catechin contents ranged from 4.1 ± 0.1 (AF-1) to 45.5 ± 1.4 mg/g dr wt (LB-5). Regarding the two other flavonoids determined, myricetin was not detected in some plants from several populations, while reached 21.2 ± 1.2 mg/g dr wt in PO-6, and quercetin glucoside varied from 3.8 ± 0.1 (CO-3) to 22.8 ± 0.8 mg/g dr wt (BT-3). Lower contents of caffeic acid were determined in bearberry leaf extracts, which varied from 1.8 ± 0.0 (IZ-1) to 7.1 ± 0.3 mg/g dr wt (SI-4). 

**Figure 4:**
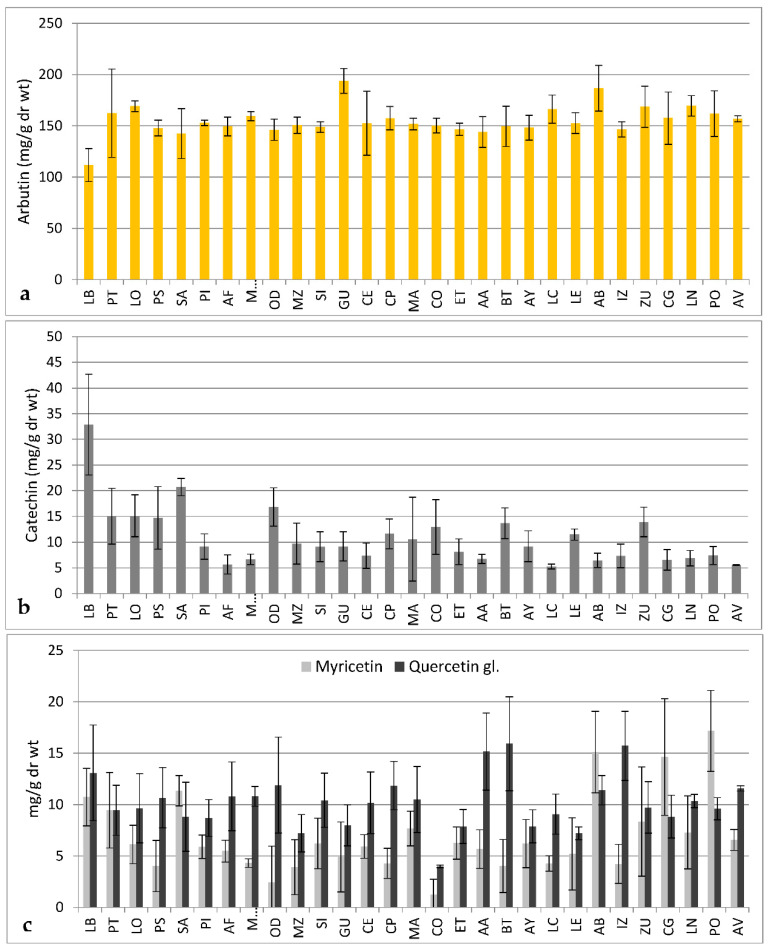
Mean contents (mg/g dr wt) of arbutin (**a**) and of the flavonoids (+) catechin (**b**), myricetin and quercetin glucoside (**c**) determined in leaves from 140 bearberry plants growing in 29 natural populations from Spain located in altitudes ranging from 534 (AV) to 1750 m a. s. l. (LB). Data are mean and standard deviation of 1–6 individuals sampled in 2016 and analyzed in triplicates.

Despite of the variation observed among individuals, significant differences among populations were also estimated for these five compounds (Kruskal–Wallis tests, *p* < 0.001, and *p* = 0.009 for caffeic acid content). Higher arbutin contents were determined, on average, in plants from AB, GU, LN, and LO natural populations (Figure 4a), which differed significantly (after Bonferroni correction) from the low contents found in plants from the LB population (186.7 ± 22.3, 193.9 ± 12.1, 169.5 ± 10.0, and 169.2 ± 5.2 mg/g dr wt front to 111.8 ± 16.0 mg/g dr wt, respectively). Again, arbutin contents of plants that had been sampled also in 2015 did not vary significantly between years (*p* = 0.821). The population LB showed in turn higher mean catechin level (Figure 4b), that significantly differed from the low mean contents of this flavonoid detected in populations AF and LC (32.8 ± 9.8 front to 5.7 ± 1.9 and 5.3 ± 0.5 mg/g dr wt, respectively). Mean myricetin and quercetin glucoside contents are shown in Figure 4c, with significant differences among populations AB, CG, LB, and PO, with higher myricetin contents (15.1 ± 4.0, 14.6 ± 5.7, 10.7 ± 2.8, and 17.2 ± 3.9 mg/g dr wt, respectively), as compared to the low levels that accumulated on average plants from populations CO and OD (1.2 ± 1.5 and 2.4 ± 3.6 mg/g dr wt, respectively), and also mean quercetin glucoside contents determined in populations AA, BT, CP, IZ, and LB (15.2 ± 3.7, 15.9 ± 4.6, 11.8 ± 2.3, 15.7 ± 3.4, and 13.1 ± 4.6 mg/g dr wt, respectively) significantly differed from that in population CO (4.0 ± 0.1 mg/g dr wt). In contrast, mean caffeic acid contents oscillated from 2.1 ± 0.2 mg/g dr wt in populations AB, CE, PT, and SA, to 3.5 ± 1.2 mg/g dr wt in population AF or 3.4 ± 2.2 mg/g dr wt in population SI; therefore, significant differences were not observed due to intrapopulation variation (averaged content 2.7 ± 0.8 mg/g dr wt). These results complement the variation on phenolic metabolites observed by Wrona et al. [20], for eight of the bearberry locations sampled in 2015 that were analyzed using the UPLC®-ESI-Q-TOF with MSE technology. 

**Figure 5:**
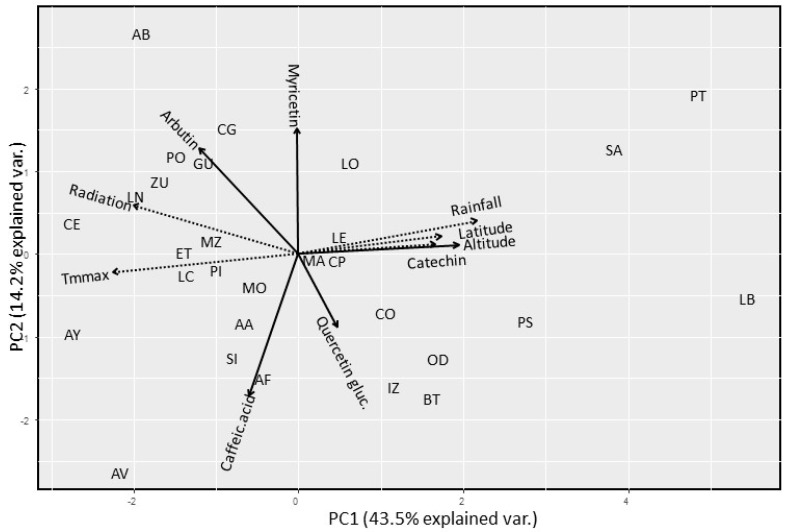
Principal components analysis of variation observed for mean contents (mg/g dr wt) in arbutin, caffeic acid, catechin, myricetin, and quercetin glucoside of 29 Spanish bearberry natural populations located at different latitudes and altitudes, and under different climatic conditions (annual rainfall, maximum mean temperature, and global radiation) that were sampled in autumn 2016.

In contrast to results obtained in analysis performed with bearberry samples from 2014 and 2015, arbutin contents determined in 2016 samples showed low but significant positive correlation with radiation (rho = 0.256, *p* = 0.002), and maximum mean temperatures (rho = 0.183, *p* = 0.030), and therefore were inversely correlated to annual rainfall values (rho = −0.265, *p* = 0.002). These differences were also associated to variation in altitude (rho = −0.192, *p* = 0.023), but this negative correlation was explained by the mentioned low arbutin contents of plants from LB population that is located at 1720 m. A similar pattern of variation was observed for myricetin contents, since higher levels of this compound were significantly associated to bearberry plants growing in locations under higher radiation levels (rho = 0.226, *p* = 0.007). The opposite pattern to that of arbutin was observed for catechin, since a positive correlation was estimated between this flavonoid and annual rainfall (rho = 0.398, *p* < 0.001), and negative correlations were accordingly detected with radiation and with maximum mean temperature (rho = −0.307, *p* < 0.001 and rho = −0.470, *p* < 0.001, respectively). This variation in climatic factors explained that both altitude and latitude affected catechin contents of bearberry leaves (rho = 0.410 and 0.490, respectively, *p* < 0.001). A principal components analysis performed with phytochemical, climatic, and geographic data corresponding to the 29 locations sampled in 2016, in which the two first components explained 60% of observed variation, corroborated these results (Figure 5). We did not detect any significant correlation between caffeic acid or quercetin glucoside contents of bearberry plants and either climatic or geographic factor, although we estimated slight but significant correlations between myricetin and quercetin glucoside contents (rho = 0.184, *p* = 0.030) and between myricetin and caffeic acid contents (rho = −0.176, *p* = 0.037) of bearberry plants.

The observed contrasting climatic patterns of variation in arbutin content of bearberry plants suggest that, although biosynthesis of this metabolite is likely increased under conditions of higher radiation and temperature, probably water scarcity is a limiting factor for *A. uva-ursi* in the Mediterranean and southern regions. Del Valle et al. [44] reported a latitudinal pattern of flavonoids accumulation of *Silene littorea* (Caryophyllaceae) in Spain, where higher flavonoids contents were determined in southern populations, since latitude correlated negatively with UV-B radiation and temperature, and positively with precipitation. Global radiation shows a clear latitudinal gradient with higher incidence in the South and East of the Iberian Peninsula, and there is also a significant orographic effect, since cloud persistence modulates the incidence of this radiation at higher altitudes. An UV-radiation map of Spain [45] referred high correlation (r > 0.9) between UV-B and global radiation data. According to this reference, bearberry populations which showed higher mean arbutin contents in our study are mostly located in areas under UV-B radiation levels of 2403–2451 J/m^2^. 

Our results for catechin variation in bearberry showed a geographical pattern (Figure 5) and agree with studies performed in other plant species from northern countries of Europe. Higher contents of specialized metabolites, such as flavonoids and anthocyanins, were reported in plants growing at higher latitudes, probably due to longer daylight periods and lower night temperatures [46]. Thereby, soluble phenolic content in *Juniperus communis* needles increased with latitude and altitude in Finland populations [47], and also 10–19% higher phenolic contents were found at a higher latitude in fruits of three *Ribes* spp. cultivars analyzed in two Finnish locations [48]. These authors related lower phenols content with higher levels of radiation and temperature. Flavonoid content in fruits of two species of *Vaccinium* (also from the family Ericaceae) showed a geographical gradient, with higher amounts of flavonoids in northern latitudes [49,50]. 

In relation to altitudinal variation, higher levels of flavonoids were determined in populations of other Ericaceae species, *Calluna vulgaris*, growing at higher altitudes [51]. Altitudinal gradients also affected the specialized metabolism of Spanish populations of *Arnica montana* [52] and *Quercus robur* [53]. The latter work referred significant positive association between the concentration of total leaf phenolics and elevation, being the gradient depending only of flavonoids, thus suggesting that these compounds drove the relationship for total phenolics. These results agree with our estimates for catechin content pattern. Finally, a rainfall gradient was associated to significant differences in polyphenols composition in an African medicinal shrub (*Myrothamnus flabellifolia*) for which metabolomic differences were congruent with the genetic structure of populations [54]. 

### 2.2. Genome Size and Sequence Data Analysis

Genome size 2C values of bearberry plants ranged from 2.50 to 3.15 pg (Table 1). Slight differences in nuclear DNA amount have been found among populations (Kruskal–Wallis χ^2^ = 87.639, d.f. = 36, *p* = 3.39 × 10^−6^). Dunn’s test with Bonferroni correction revealed statistically significant differences between: AL and SI populations (Z = −3.972, p = 0.024), LE and SI (Z = −4.196, *p* = 0.009), and SE and SI (Z = −4.236, *p* = 0.008). The population of Pontils (PO) is the type locality of *A. uva-ursi* var. *crassifolius*, which was subsequently combined as *A. uva-ursi* subsp. *crassifolius*, and currently not considered taxonomically (see introduction), has an intermediate value (2.88 pg), within the range of values obtained in other populations of the species. This is the first extensive populational study of nuclear DNA amount in the species and the whole genus, since the only available information to date comes from one Balkan population of the same taxon [55]. The 2C value there reported, 2.49 pg, is placed just at the lower limit of the range of variation of the here investigated dataset. No correlation between genome size values and population altitude was found. The DNA amount of individuals showed a weak positive significant correlation with the arbutin content when data from 158 bearberry plants were compared (rho = 0.187, *p* = 0.018), while no other significant correlation between genome size and other variables was found. This result is in agreement with the reported increases in the production of specialized metabolites that are generally observed in medicinal and aromatic plants after polyploidization [29]. 

**Figure 6:**
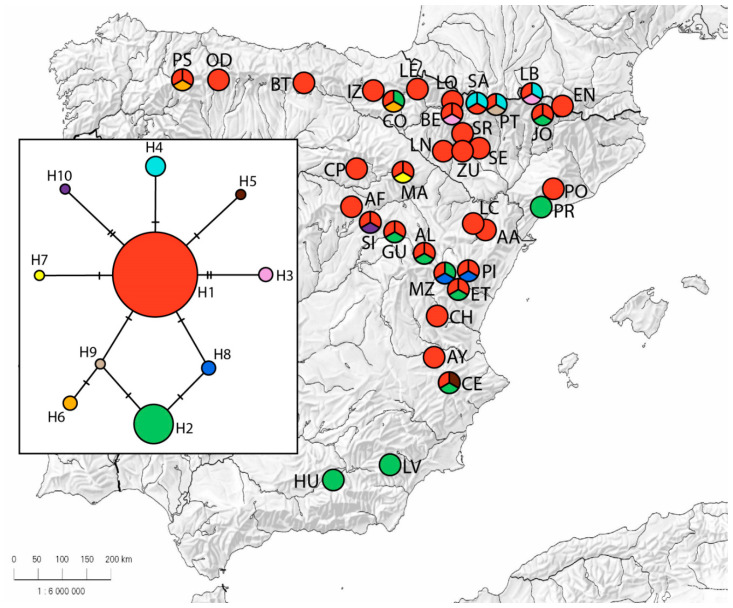
Geographical distribution of pDNA haplotypes on Iberian populations of *Arctostaphylos uva-ursi*. Population codes correspond to those in Table S1, and pie charts represent the percentage of individuals showing each haplotype in each population. In the rectangle, the statistical parsimony network showing the evolutionary relationships of the ten plastid haplotypes found in *A. uva-ursi* populations is represented. Each stripe along the lines connecting the haplotypes indicates one mutational step in the pDNA regions sequenced in this study.

Newly produced sequences for rpl32-trnL and psbE-petN intergenic spacers were aligned in two matrices (respectively containing 566 and 874 bp) which were concatenated in a single matrix of 1440 bp. Moderate levels of variation in these plastid regions were detected (six nucleotide substitutions and four indels). Ten different haplotypes were found with a total haplotype diversity (Hd) of 0.468 (Table 1). Most populations (21) contained only one haplotype, while five of them exhibited some diversity among individuals. The most abundant haplotype 1 showed the highest frequency (71.4%), followed by haplotype 2 (15.2%), whereas the rest of them showed much lower frequencies (0.04–0.01%). The geographic distribution of haplotypes is shown in Figure 6. Haplotype 1 was found in 32 populations—it was fixed in 18 of them—distributed across all sampled regions except for the southernmost localities. Haplotype 2 was found in ten populations and was the exclusive haplotype in the two southernmost populations (HU and LV) as well as in PR. Haplotype 4 was private from three populations from the Pyrenees, while haplotype 8 appeared in two populations from eastern Iberian Peninsula. Regarding the evolutionary relationships shown in the parsimony network (Figure 6), most of the haplotypes are connected by one or two mutation steps. Haplotype 1 occupies a central position, with seven haplotypes being connected to it. 

A Mantel test between the pDNA genetic and chemical pairwise distance matrices showed weak but significant correlation (r = 0.301; *p* = 0.048) among genetic differentiation and the concentration of the five chemical components measured for the 2016 data set. The Mantel test did not reveal any significant correlation between genetic and geographic distance among the 25 populations sampled in 2016 (r = 0.103, *p* = 0.207) or between the chemical and geographic pairwise distances among these 25 populations (r = 0.191, *p* = 0.050). The phytochemical variability reported here for bearberry wild populations across Spain is therefore complemented by moderate levels of pDNA haplotype variation, being the north–south genetic differentiation found in bearberry populations comparable to phylogeographic patterns found in other plants (e.g., [56,57,58]. All this homogeneity is expected in plants with low reproductive capacity [59], as *A. uva-ursi*, which exhibits poor germination rates and high mortality rates of young seedlings [4]. Nevertheless, the modest genetic differentiation showed by pDNA correlated with biochemical pairwise distances estimated from profiles of five phenolic compounds. This pattern of genetic and phytochemical variation (which, as mentioned, was not associated to geographical distances), as well as the weak but significant correlation between genome size and arbutin content, may indicate that natural genomic variability affects the yield of phenolic compounds in *A. uva-ursi*. However, hypervariable molecular markers in a more extensive population sampling are necessary to corroborate these phylogeographic patterns and the association between genetic and biochemical variability.

## 3. Materials and Methods

### 3.1. Plant Material

For phytochemical analysis, a total of 249 bearberry plants growing in 42 populations were sampled in autumn 2014, 2015, and 2016, being representative of the natural distribution of this plant species in the Iberian Peninsula (Figure 1 and Table S1). First, in autumn 2014, we collected terminal shoots from 80 plants in ten Spanish populations (AG, AN, BA, LI, LO, PE, PÁ, SR, SC, and SE) located in a relatively small area (80 × 50 km approximately) in North Spain but in a wide range of altitude (424–1410 m a. s. l.). Second, in autumn 2015, we collected 48 plants from six of these populations (AG, LI, LO, PA, SE, and SR) and 46 bearberry plants from six populations located at lower latitudes (AL, CH, ET, HU, LV, and PI). For each population, we sampled eight plants separated at least 5 m, except in HU, where we found only six individuals. Third, in autumn 2016, a total of 140 plants were sampled in 29 different locations (1–6 individuals from each) including 26 new Spanish localities (IZ, CE, BT, MO, AA, GU, SI, AF, SA, PT, AB, CG, OD, PS, LB, CO, LE, MA, CP, PO, MZ, AV, LC, AY, LN, and ZU). Our sampling spans an altitude range from 534 to 1750 m. Altogether, the 42 populations represent a wide range of climatic conditions (Table S1): maximum mean temperature showed 2-fold variation (13–26 °C) and annual precipitation oscillated from 399 (ZU) to 1589 mm (PT). Global radiation ranged from 4.2 to 5.1 kWh/m^2^d. Six to ten terminal shoots (15–20 cm long) were collected for each plant to obtain 6–15 g of healthy leaves, which were subsequently excised and dried at 60 °C to constant weight (3–4 days). After dry weight determinations, leaves were manually homogenized in a mortar and stored at 4 °C until analyzed. This plant material was employed for total phenols and HPLC determinations.

Leaf materials for flow cytometric assessments were obtained from fresh leaves of 178 individuals belonging to a total of 37 populations (Table 1). Samples were obtained from 33 of the populations mentioned above, and from four new populations (BE, JO, and PR in Spain, and EN in Andorra) where leaves were collected in spring 2017, and therefore were not included in the phytochemical analysis (Figure 1 and Table S1). From one to six individuals per population were measured in duplicates. Leaf materials for DNA extraction were dried in silica-gel and stored at room temperature. DNA diversity analysis were performed in a total of 105 plants from 35 locations (Table 1).

Plant vouchers of the 46 bearberry populations studied are deposited in the herbarium BC, of the Institut Botànic de Barcelona, in the herbarium BCN, of the Centre de Documentació de Biodiversitat Vegetal, Universitat de Barcelona or in the herbarium JACA, of the Instituto Pirenaico de Ecología (CSIC).

### 3.2. Preparation of Leaf Extracts and Total Phenols Determination

Three replicates of bearberry leaf extracts were prepared by using 50 mg of dried sample and 10 mL of 80% methanol. Tubes were incubated for 30 min in an ultrasonic bath, and then extracts were filtered (0.45 mm) and stored at 4 °C until analyzing. Total phenolic content was determined in the extracts prepared from samples collected in 2014 and 2015. We followed a Folin–Ciocalteu method [60,61] with slight modifications: to 0.1 mL of extract, 0.4 mL of methanol (80%), 0.5 mL of Folin–Ciocalteu reagent and 8 mL of ultrapure water were added. After 5 min in an ultrasonic bath, 1 mL of Na_2_CO_3_ 20% (w/v) was added. The samples were left in the dark for 30 min before measuring absorbance at 760 nm using a UV–visible spectrophotometer (CARY 50 BIO, Varian, Agilent Technologies). Results were expressed in gallic acid equivalents (GAE); that is, mg gallic acid/g dr wt, using a gallic acid standard curve (40–340 μg/g). 

### 3.3. Arbutin and Other Phenolic Metabolites Determination

Phenolic contents of bearberry leaf methanolic extracts were quantified by RP-HPLC using a HPLC-UV/Vis system (LaChrom Merck Hitachi L-7400) with a Kinetex 5 µm-EVO C18 (250 mm × 4.6 mm) column, and absorbance determined at 280 nm. Extracts from samples collected in 2014 and 2015 were diluted 1:10 (v/v) and injected with a mobile phase of methanol and acetonitrile. The gradient program consisted of 0–5 min, 25% of methanol; 5–6 min, 100%; 6–10 min, 100%; and 10–20 min, again 25% of methanol. Flow rate was 1 mL/min and the injection volume was 20 µL. In these conditions, the retention time of arbutin was 2.6 min, enabling us to determine the concentration of this compound using a calibration curve established with 6 dilutions of a standard, from 40 to 340 µg/g, with a correlation coefficient of r = 0.9997. Extracts from samples collected in 2016 were analyzed using a mobile phase of methanol and water with the following gradient program: 0–5 min, 10% of methanol; 5–6 min, 20%; 6–10 min, 20%; 10–11 min, 30%; 11–15 min, 30%; 15–16 min, 40%; 16–20 min, 40%; 20–21 min, 50%; 21–25 min, 50%; 25–26 min, 60%; 26–30 min, 60%; 30–31 min, 10%; and 31–36 min, again 10% of methanol. In these conditions, we determined arbutin and other 4 phenolic compounds: caffeic acid, catechin, myricetin, and quercetin-3-O-glucopyranoside using the corresponding calibration curves established with 5 dilutions of standards (25–500 µg/mL), which showed correlation coefficients of 0.9992 for catechin (retention time 12.0 min), 0.9924 for caffeic acid (retention time 13.4 min), 0.9981 for quercetin-3-O-glucoside (retention time 23.4 min), and 0.9997 for myricetin (retention time 25.1 min). 

Methanol and acetonitrile were in HPLC grade and purchased from Panreac (Barcelona, Spain). Standards (purity ≥ 98%) of arbutin, caffeic acid, and gallic acid, as well as the Folin–Ciocalteu reagent were purchased from Sigma-Aldrich (Barcelona, Spain). Catechin and quercetin-3-O-glucopyranoside standards were purchased from Extrasynthese (Genay Cedex, France), while myricetin was purchased from Alfa Aesar (Karlsruhe, Germany).

Results of chemical determinations were analyzed using SPSS v. 25 software and R 3.5.2. After testing data for normality and homoscedasticity, we performed analysis of variance of different sets of data and means separation tests (Tukey and Tamhane) or non-parametric tests as Kruskal–Wallis, and Dunn multiple comparison test with Bonferroni correction. We also estimated correlation coefficients (Pearson or Spearman depending if data followed a normal distribution) between variables, including genome size. 

### 3.4. Genome Size Estimates and DNA Sequencing

Genome size of bearberry plants was estimated by flow cytometry at the Centres Científics i Tecnològics, Universitat de Barcelona (CCiTUB) following the procedures explained in Pellicer et al. [62]. *Petunia hybrida* Vilm. ‘PxPc6’ (2C = 2.85 pg) was used as internal standard. Seeds of the standard were provided by the Plateforme de cytométrie d’Imagerie-Gif, CNRS—I2BC (Gif-sur-Yvette, France). Nuclear DNA contents (2C) were calculated by multiplying the known DNA content of the standard by the quotient between the peak positions (mode) of the target species and the standard in the histogram of fluorescence intensities, assuming a linear correlation between the fluorescent signals from the stained nuclei of the unknown specimen, the known internal standard and the DNA amount [63].

Approximately 20 mg of silica-dried leaf tissue was used for DNA extraction using a CTAB protocol [64] with minor modifications. Quality of total DNA was checked with a NanoDrop 1000 spectrophotometer (ThermoScientific, Wilmington, DE, USA). The plastid intergenic regions rpl32-trnL and psbE-petN, as well as the nuclear ribosomal DNA region ITS, were amplified and sequenced for three individuals per population. Sequences of ITS presented no variability within the 105 analyzed individuals, excepting for a few positions showing intragenomic nucleotide polymorphisms. Therefore, this nuclear ribosomal DNA region was excluded from further analysis. Other plastid DNA regions (i.e., ndhF; ndhF-rpl32; psbA-trnH; psbD-trnT; rps16; and trnL-trnF) were also tested but they were discarded due to low variability or sequencing problems. Amplification procedure was performed as described in Vitales et al. [65]. Direct sequencing of the amplified DNA segments was performed with Big Dye Terminator Cycle Sequencing v 3.1 (PE Biosystems, Foster City, California, USA) at the Unitat de Genòmica, CCiTUB, on an ABI PRISM 3700 DNA analyzer (PE Biosystems). The rpl32-trnL and psbE-petN sequences were assembled with BioEdit version 7.1.3.0 [66], aligned with ClustalW Multiple Alignment v1.4 [67] and adjusted manually. GenBank accession numbers are provided in Table S2.

### 3.5. Genome Size and Genetic Analysis

A non-parametric Kruskal–Wallis test was performed to check for statistically significant differences in 2C values among populations. The Dunn multiple comparison test was also performed to determine which populations showed significant differences between them. The Bonferroni correction was applied to minimize the type I error (false positive). 

Genetic diversity parameters (polymorphic sites, parsimony informative sites, number of haplotypes, and total haplotype diversity) were estimated using DnaSP v6 [68]. Plastid haplotypes were determined in a combined data set that included both the rpl32-trnL and psbE-petN regions. Indels were codified with FastGap v.1.2 [69] and treated as single-mutation events. The evolutionary relationships among haplotypes were inferred based on a parsimony network constructed using TCS [70] as implemented in PopArt [71]. The maximum number of differences resulting from single substitutions among haplotypes was calculated with 95% confidence limits.

To analyze the relationship among genetic and chemical data a Mantel test was performed. First, we estimated the phenotypic distance among 25 populations using data from bearberry plants collected in 2016, which had been measured for five phenolic components (i.e. arbutin, catechin, caffeic acid, quercetin, and myricetin). We used R Commander to standardize these data [72], and subsequently, to calculate the chemical distance derived from the standardized matrix with the function “Dist” on R based on Euclidean distances. Second, a Nei’s population genetic pairwise distance matrix was calculated between the 25 populations using DnaSP v6. Finally, we calculated the pairwise geographic distances among these populations using the package “geodist” in R. Pairwise correlations between distance matrices were computed using a Mantel test with 10,000 permutations with the function mantel available in the R package “vegan” [73]. 

## 4. Conclusions

All analyzed bearberry plants showed arbutin contents higher than 7%; therefore, this plant material is appropriate for herbal preparations. Our analysis revealed higher arbutin contents than those (up to 9%) referred by Parejo et al. [40], who analyzed four populations located in the north-eastern Pyrenees at 1580–2030 m and detected low differences in arbutin content among populations. Furthermore, most of the values determined herein are in the range of the plant material selected for cultivation in Poland [74] and in agreement with the high arbutin content of Spanish bearberry leaf samples reported by Sonnenschein and Tegtmeier [42] when selecting plants for cultivation in Germany. Elite genotypes of *A. uva-ursi* could be vegetatively propagated, but most of the obtained clones failed during field establishment, which limited exploitation of these resources. We also described the existing variation in the content of three flavonoids (catechin, myricetin, and quercetin glucoside), as well as of caffeic acid, among Spanish bearberry populations, and how climatic factors (mainly global radiation and rainfall), which correlated to latitudinal and altitudinal gradients, affect that variation. At the same time, despite the low levels of haplotype and genome size variability determined in Iberian *A. uva-ursi*, genetic and cytogenetic differentiation of populations was weakly but significantly associated to phytochemical diversity. Overall, these results highlight that the effect of both genetic and abiotic factors must be considered to explain intraspecific phytochemical variability found in plants from wild populations.

## Figures and Tables

**Table 1:** Genome size (GS) values and haplotypes of 37 and 35 bearberry populations respectively analyzed for those variables. The number of individuals studied for genome size assessment [N**_(GS)_**] and for haplotype analysis [N**_(H)_**] are also indicated.

Population Code	N _(GS)_	GS ± SD	N _(H)_	Haplotypes
AA	5	2.66 ± 0.28	3	H1
AF	5	2.87 ± 0.10	3	H1
AL	5	2.53 ± 0.08	3	H1, H2
AV	1	2.87	-	-
AY	5	2.66 ± 0.07	3	H1
BE	5	2.70 ± 0.05	3	H1, H3
BT	5	2.92 ± 0.08	3	H1
CE	5	2.89 ± 0.27	3	H1, H2, H5
CH	5	2.85 ± 0.10	3	H1
CO	6	2.79 ± 0.09	3	H1, H2, H6
CP	6	2.87 ± 0.09	3	H1
EN	5	2.67 ± 0.09	3	H1
ET	5	2.81 ± 0.12	3	H1, H2
GU	5	2.83 ± 0.06	3	H1, H2
HU	5	2.74 ± 0.04	3	H2
IZ	5	2.78 ± 0.03	3	H1
JO	5	2.61 ± 0.16	3	H1, H2
LB	5	2.76 ± 0.07	3	H1, H3, H4
LC	5	2.74 ± 0.25	3	H1
LE	5	2.50 ± 0.16	3	H1
LN	5	3.09 ± 0.35	3	H1
LO	5	2.81 ± 0.11	3	H1
LV	5	2.68 ± 0.11	3	H2
MA	6	2.86 ± 0.05	3	H1, H7
MO	1	2.83	-	-
MZ	5	2.87 ± 0.17	3	H1, H2, H8
OD	5	2.82 ± 0.10	3	H1
PI	5	2.86 ± 0.12	3	H1, H8
PO	5	2.88 ± 0.35	3	H1
PR	5	2.79 ± 0.18	3	H2
PS	5	2.82 ± 0.08	3	H1, H6
PT	5	2.72 ± 0.18	3	H1, H4, H9
SA	2	3.13 ± 0.53	3	H1, H4
SE	5	2.53 ± 0.11	3	H1
SI	5	3.15 ± 0.33	3	H1, H10
SR	5	2.66 ± 0.07	3	H1
ZU	6	2.84 ± 0.19	3	H1

## Supplementary Materials

The following are available online at https://www.mdpi.com/2223-7747/9/9/1250/s1, Table S1: Location and climatic conditions of 46 bearberry populations sampled across four years (2014–2017) in the Iberian Peninsula. Herbarium voucher codes are also provided. Chemical analysis was performed using plant material from 42 populations sampled from 2014 to 2016, while 4 populations sampled in 2017 were used only for genetic and cytogenetic studies. Table S2: GenBank accession numbers of the plastid intergenic regions rpl32-trnL and psbE-petN, as well as the nuclear ribosomal DNA region ITS, of 105 bearberry plants from the Iberian Peninsula. Figure S1. Example of chromatograms obtained for the 5 phenolic standards: A, arbutin; C, catechin; F, caffeic acid; QG, quercetin-o-glucoside; and M, myricetin.
