# Supplementary material for: Phenolic Compounds Content and Genetic Diversity at Population Level across the Natural Distribution Range of Bearberry (Arctostaphylos uva-ursi, Ericaceae) in the Iberian Peninsula
Source: Plants (Basel). 2020 Sep 22;9(9):1250. doi: 10.3390/plants9091250 (PMC7570137; doi:10.3390/plants9091250)

Table S1: Location and climatic conditions of 46 bearberry populations sampled across four years (2014–2017) in the Iberian Peninsula. Herbarium voucher codes are also provided. Chemical analysis were performed using plant material from 42 populations sampled from 2014 to 2016, while 4 populations sampled in 2017 were used only for genetic and cytogenetic studies.

Population, Location	Code	Province	Latitude (N)	Longitude	Altitude (m)	Annual precipitation (mm)*	Range of mean temperatures (°C)*	Global Radiation (kWh/m2d)^	Number of plants	Years sampled	Herbarium voucher code
Izki, Arraia-Maeztu	IZ	Álava	42º 42'	(W) 2º 25'	761	747	4–19	4.2	5	2016	BC 976486
Caudete	CE	Albacete	38º 44'	(W) 1º 02'	1125	417	8–24	5.1	5	2016	BC 976493
Sierra de María, Los Vélez	LV	Almería	37º 41’	(W) 2º 12’	1369	439	5–22	5.1	6	2015	BC 963387
Basconcillos del Tozo	BT	Burgos	42º 42'	(W) 3º 59'	920	719	3–18	4.3	5	2016	BC 976490
La Puebla de Alcolea	AA	Castellón	40º 43'	(W) 0º 05'	992	498	5–22	4.6	5	2016	BC 976497
El Toro	ET	Castellón	39º 56'	(W) 0º 44'	998	506	4–21	4.8	8	2015, 2016	BC 963348
Morella	MO	Castellón	40º 39'	(W) 0º 04'	1154	570	4–20	4.6	1	2016	BC 976496
Pina de Montalgrao	PI	Castellón	40º 01'	(W) 0º 37'	1278	522	4–21	4.8	8	2015, 2016	BC 963350
Sierra de Huétor	HU	Granada	37º 16’	(W) 3º 30’	1354	550	7–26	5.1	8	2015	BC 963386
Albendiego	AF	Guadalajara	41º 12'	(W) 3º 03'	1270	535	2–20	4.6	5	2016	BC 976499
Corduente	GU	Guadalajara	40º 51'	(W) 2º 00'	1140	485	2–20	4.6	5	2016	BC 976483
Siguenza	SI	Guadalajara	41º 04'	(W) 2º 34'	1140	477	3–21	4.7	5	2016	BC 976481
Alcubierre	AB	Huesca	41º 43'	(W) 0º 27'	780	415	4–21	4.9	5	2016	BC 106836
Agüero	AG	Huesca	42º 21'	(W) 0º 47'	738	647	3–19	4.7	8	2014, 2015	JACA 42580
Aniés	AN	Huesca	42º 18'	(W) 0º 34'	881	577	3–19	4.8	8	2014	JACA 42993
Barbastro	BA	Huesca	42º 02'	(E) 0º 04'	424	490	5–22	4.9	8	2014	JACA 237358
Colungo	CG	Huesca	42º 10'	(W) 0º 04'	620	585	3–20	4.7	5	2016	JACA 105748
Lierta	LI	Huesca	42º 12'	(W) 0º 28'	590	474	5–23	4.9	8	2014, 2015	JACA 71101
Sierra de Loarre	LO	Huesca	42º 20'	(W) 0º 36'	1401	640	3–19	4.8	8	2014, 2015, 2016	BC 963389
Pico del Águila, Arguis	PA	Huesca	42º 18'	(W) 0º 24'	1410	633	2–18	4.7	8	2014, 2015	JACA 71033
Perarrúa	PE	Huesca	42º 16'	(E) 0º 20'	687	651	4–20	4.7	8	2014	JACA 175415
Panticosa	PT	Huesca	42º 45'	(W) 0º 13'	1692	1589	−1–13	4.2	5	2016	BC 963351
Sallent de Gállego	SA	Huesca	42º 46'	(W) 0º 19'	1311	1034	0–15	4.2	2	2016	BC 963357
Santa Cruz de la Serós	SC	Huesca	42º 31'	(W) 0º 40'	820	703	2–18	4.6	8	2014	JACA 42643
Salto de Roldán, Nueno	SR	Huesca	42º 15'	(W) 0º 23'	1016	586	3–19	4.8	8	2014, 2015	BC 963352
Otero Dueñas	OD	León	42º 47'	(W) 5º 43'	1152	690	3–18	4.5	5	2016	BC 976488
Puerto de Somiedo, Cabrillanes	PS	León	42º 59'	(W) 6º 13'	1340	905	2- 16	4.3	5	2016	BC 976489
Puerto de La Bonaigua, Baqueira	LB	Lleida	42º 40'	(E) 0º 57'	1750	1083	0–16	4.3	5	2016	BC 963397
Sierra de Codés, Torralba del Río	CO	Navarra	42º 37'	(W) 2º 20'	1042	702	4–20	4.2	6	2016	BC 976487
Sierra de Leyre, Yesa	LE	Navarra	42º 38'	(W) 1º 10'	796	831	4–20	4.5	5	2016	BC 963356
Cabrejas del Pinar	CP	Soria	41º 46'	(W) 2º 51'	1112	580	2–19	4.6	6	2016	BC 976491
Matalebreras	MA	Soria	41º 49'	(W) 2º 04'	1070	563	2–19	4.6	6	2016	BC 976492

Pontils	PO	Tarragona	41º 28'	(E) 1º 22'	583	645	6–22	4.7	6	2016	BC 963398
Albarracín	AL	Teruel	40º 23'	(W) 1º 24'	1337	498	2–20	4.7	8	2015	BC 963353
Aguaviva	AV	Teruel	40º 47'	(W) 0º 09'	534	465	5–22	4.6	1	2016	BC 976494
La Cerollera	LC	Teruel	40º 51'	(W) 0º 02'	842	546	4–21	4.6	5	2016	BC 976495
Manzanera	MZ	Teruel	40º 02'	(W) 0º 49'	1144	494	4–21	4.8	5	2016	BC 976485
La Hunde, Ayora	AY	Valencia	39º 04'	(W) 1º 14'	910	466	7–24	5.0	5	2016	BC 976482
Pico del Remedio, Chelva	CH	Valencia	39º 46'	(W) 0º 59'	984	401	7–24	4.9	8	2015	BC 963349
Bardena Negra, Ejea	LN	Zaragoza	42º 04'	(W) 1º 20'	601	513	4–24	4.8	5	2016	BC 976484
Santa Eulalia de Gállego	SE	Zaragoza	42º 17'	(W) 0º 45'	538	578	4–20	4.8	8	2014, 2015	BC 963354
Zuera	ZU	Zaragoza	41º 56'	(W) 0º 56'	681	399	5–23	4.9	5	2016	BC 976498
Engolasters	EN	Andorra	42º 31’	(E) 1º 34’	1616	1077	0–15		5	2017	BC 963399
Jou	JO	Lleida	42º 36’	(E) 1º 07’	1388	943	2–18	4.4	5	2017	BC 963355
Berdún	BE	Huesca	42º 34'	(W) 0º 49'	611	781	3–19	4.4	5	2017	BCN 101067
Prades	PR	Tarragona	41º 19’	(E) 1º 03’	1060	728	4–20	4.7	5	2017	BC 963390
Total						**http://es.climate-data.org/*	*^http://adrase.com*		269		

Table S2: GenBank accession numbers of the plastid intergenic regions rpl32-trnL and psbE-petN, as well as the nuclear ribosomal DNA region ITS, of 105 bearberry plants from the Iberian Peninsula.

Specimen code	ITS	rpl32-trnL	psbE-petL
AF_1	MN663163	MN695046	MN695151
AF_2	MN663164	MN695047	MN695152
AF_3	MN663165	MN695048	MN695153
AL_1	MN663166	MN695049	MN695154
AL_2	MN663167	MN695050	MN695155
AL_3	MN663168	MN695051	MN695156
AA_1	MN663169	MN695052	MN695157
AA_2	MN663170	MN695053	MN695158
AA_5	MN663171	MN695054	MN695159
IZ_1	MN663172	MN695055	MN695160
IZ_2	MN663173	MN695056	MN695161
IZ_3	MN663174	MN695057	MN695162
LV_1	MN663175	MN695058	MN695163
LV_2	MN663176	MN695059	MN695164
LV_3	MN663177	MN695060	MN695165
HU_4	MN663178	MN695061	MN695166
HU_1	MN663179	MN695062	MN695167
HU_6	MN663180	MN695063	MN695168
AY_2	MN663181	MN695064	MN695169
AY_3	MN663182	MN695065	MN695170
AY_5	MN663183	MN695066	MN695171
LB_1	MN663184	MN695067	MN695172
LB_2	MN663185	MN695068	MN695173
LB_5	MN663186	MN695069	MN695174
BT_1	MN663187	MN695070	MN695175
BT_2	MN663188	MN695071	MN695176
BT_3	MN663189	MN695072	MN695177
BE_1	MN663190	MN695073	MN695178
BE_2	MN663191	MN695074	MN695179
BE_3	MN663192	MN695075	MN695180
CE_1	MN663193	MN695076	MN695181
CE_4	MN663194	MN695077	MN695182
CE_5	MN663195	MN695078	MN695183
CH_1	MN663196	MN695079	MN695184
CH_2	MN663197	MN695080	MN695185
CH_3	MN663198	MN695081	MN695186
CO_1	MN663199	MN695082	MN695187
CO_2	MN663200	MN695083	MN695188
CO_3	MN663201	MN695084	MN695189
CP_1	MN663202	MN695085	MN695190
CP_2	MN663203	MN695086	MN695191
CP_3	MN663204	MN695087	MN695192
EN_1	MN663205	MN695088	MN695193
EN_2	MN663206	MN695089	MN695194
EN_3	MN663207	MN695090	MN695195
LC_1	MN663208	MN695091	MN695196
LC_4	MN663209	MN695092	MN695197
LC_5	MN663210	MN695093	MN695198
JO_3	MN663211	MN695094	MN695199
JO_4	MN663212	MN695095	MN695200
JO_5	MN663213	MN695096	MN695201
LE_1	MN663214	MN695097	MN695202
LE_2	MN663215	MN695098	MN695203

LE_3	MN663216	MN695099	MN695204
LN_1	MN663217	MN695100	MN695205
LN_2	MN663218	MN695101	MN695206
LN_3	MN663219	MN695102	MN695207
LO_1	MN663220	MN695103	MN695208
LO_2	MN663221	MN695104	MN695209
LO_5	MN663222	MN695105	MN695210
MA_1	MN663223	MN695106	MN695211
MA_2	MN663224	MN695107	MN695212
MA_3	MN663225	MN695108	MN695213
GU_1	MN663226	MN695109	MN695214
GU_2	MN663227	MN695110	MN695215
GU_4	MN663228	MN695111	MN695216
MZ_1	MN663229	MN695112	MN695217
MZ_2	MN663230	MN695113	MN695218
MZ_4	MN663231	MN695114	MN695219
OD_1	MN663232	MN695115	MN695220
OD_4	MN663233	MN695116	MN695221
OD_5	MN663234	MN695117	MN695222
PT_1	MN663235	MN695118	MN695223
PT_4	MN663236	MN695119	MN695224
PT_5	MN663237	MN695120	MN695225
PI_1	MN663238	MN695121	MN695226
PI_2	MN663239	MN695122	MN695227
PI_4	MN663240	MN695123	MN695228
PO_1	MN663241	MN695124	MN695229
PO_2	MN663242	MN695125	MN695230
PO_5	MN663243	MN695126	MN695231
PR_1	MN663244	MN695127	MN695232
PR_5	MN663245	MN695128	MN695233
PR_6	MN663246	MN695129	MN695234
PS_1	MN663247	MN695130	MN695235
PS_2	MN663248	MN695131	MN695236
PS_3	MN663249	MN695132	MN695237
SA_1	MN663250	MN695133	MN695238
SA_2	MN663251	MN695134	MN695239
SA_3	MN663252	MN695135	MN695240
SE_1	MN663253	MN695136	MN695241
SE_2	MN663254	MN695137	MN695242
SE_5	MN663255	MN695138	MN695243
SI_1	MN663256	MN695139	MN695244
SI_2	MN663257	MN695140	MN695245
SI_4	MN663258	MN695141	MN695246
SR_2	MN663259	MN695142	MN695247
SR_3	MN663260	MN695143	MN695248
SR_5	MN663261	MN695144	MN695249
ET_1	MN663262	MN695145	MN695250
ET_2	MN663263	MN695146	MN695251
ET_3	MN663264	MN695147	MN695252
ZU_1	MN663265	MN695148	MN695253
ZU_2	MN663266	MN695149	MN695254
ZU_3	MN663267	MN695150	MN695255

Figure S1. Example of chromatograms obtained for the 5 phenolic standards: A, arbutin; C, catechin; F, caffeic acid; QG, quercetin-o-glucoside; and M, myricetin.

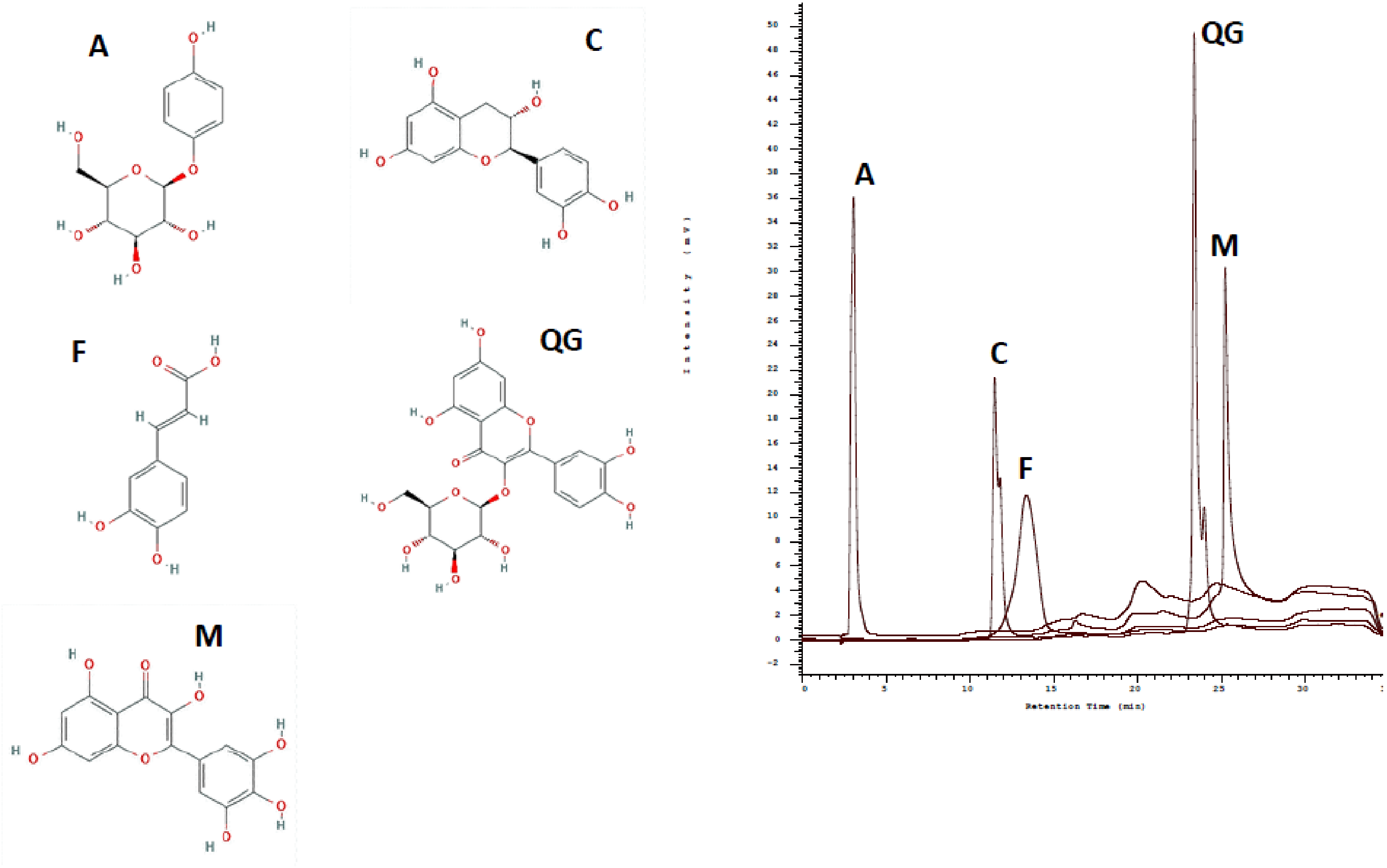

Supplement: Supplementary file 1 [file plants-09-01250-s001.pdf]
